# Testing the Ability of Dogs to Detect Different Odor Concentrations of the Carolina Anole (*Anolis carolinensis*) in Japan

**DOI:** 10.3389/fvets.2020.590834

**Published:** 2020-10-30

**Authors:** Megumi Fukuzawa, Koki Shibata

**Affiliations:** Department of Animal Science and Resources, College of Bioresource Sciences, Nihon University, Fujisawa, Japan

**Keywords:** alien species, detection, dog, judging eradication, training

## Abstract

The Carolina anole (*Anolis carolinensis*) is regarded as a problem in the Ogasawara Islands. The decision to use eradication measures depends on the limit of detection at low densities. We tested the ability of two dogs to discriminate the odor of anole to assess the possibility of using dogs to detect anoles at low densities. The two dogs were trained to discriminate the basic target odor concentration (512 anoles/ha) on 10-g coconut peat sachets. When they reached 100% accuracy, they were tested at different odor concentrations (densities of 385, 256, 128, 26, and 3 anoles/ha). During training, both dogs achieved 100% accuracy after 2 daily sessions in only 2 days. They were able to select the positive odor concentration sachet, and their accuracy was from 75 to 100%. We believe that testing using soil from sites of high anole high density and at the limit of detection in the Ogasawara Islands will be useful.

## Introduction

The Ogasawara Islands consist of more than 30 archipelagos in 5 groups, with a total land area of 7,939 hectares. These islands' ecosystems clearly reflect the evolutionary processes of many endemic species, and the islands were registered as a World Heritage Site in 2011 (1). On the otherhand, the native insect community in Ogasawara has declined greatly in all areas owing to predation by the anole. The Japanese Ministry of the Environment, therefore, listed the Carolina anole as an invasive alien species in Japan in June 2005, barring its spread to new islands and setting natural regeneration zones in remaining habitats of native insects. The anole is controlled by setting polypropylene adhesive traps around tree trunks to capture them, and by the erection of barrier fences and electric fences. Adhesive traps are effective except where numbers are low, and therefore do not allow us to judge whether a population has been eliminated or not. Since the Carolina anole (*Anolis carolinensis*) was found on Anijima island in 2013, intensive measures to eradicate them have been in progress (2). Although the anole population density on Anijima is at most only 1/10 of that on Chichijima, it is difficult to judge the success of eradication when the density is so low.

The keen olfactory ability of dogs is used in the conservation of various species [e.g., (3–5)], and is considered effective in judging eradication in areas of low density. For example, Kretser et al. (6) concluded that scat-detection dogs were effective at locating moose (*Alces alces*) scat in areas of low population density in New York state, USA. Statham et al. (7) also reported that dog-handler teams are a promising survey tool to detect the presence of blunt-nosed leopard lizard (*Gambelia sila*) and to help increase sample sizes of scats necessary for subsequent DNA analysis for research and conservation purposes. Since the Ogasawara Islands are a World Heritage Sites, access to uninhabited islands is restricted to both people and detection dogs. Not only are detection dogs required to survey huge areas daily, but they are needed with certain considerations (e.g., cultivate cost, training period). Even if the dog has high olfactory detection ability, we cannot use as a detection dog unless it has a physical body that can withstand fieldwork. Ironically, gaining experience will strengthen (stabilize) the dog's detection ability, however, the environment to be detected is often severe for dogs, and their physical level also decreases with age. Therefore, it may be more practical to carry out discrimination tests on soil samples rather than *in situ*. If we are able to confirm the detection ability of dogs in indoor, then it may reduce various risks to dogs when these detecting performed under adverse conditions.

We tested the ability of two dogs to discriminate the odor of anoles to assess the possibility of using dogs to detect anoles at low densities.

## Materials and Methods

### Dogs

We trained two healthy adult female dogs (German Shepherd, 43 months, 25.8 kg; Labrador Retriever, 52 months, 23.6 kg). They had previously been trained to detect odors of Carolina anoles' bodies and excrement/urine (unpublished data), first learning to recognize body odor and then excrement/urine odor. The dogs were kept in a familiar kennel at the university during the day, where they were allowed contact with the outdoors and with humans at all times. Each dog was trained by a different handler. The German Shepherd's handler was a woman who had been a handler for nearly 2 years with GS, and the Labrador's handler was a familiar male person with both dogs, but this was his first time to handle LR.

### Target Odor

**Figure 1:**
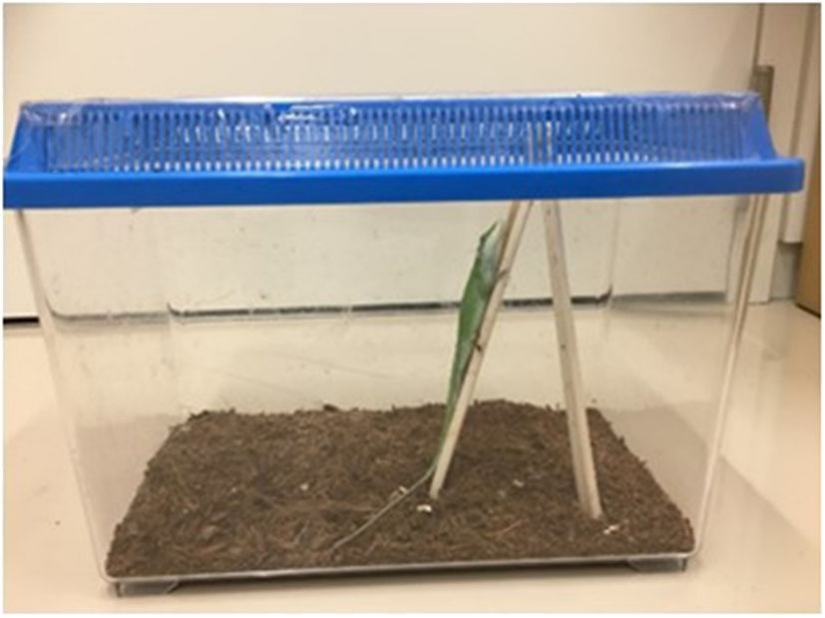
An anole in its cage.

**Figure 2:**
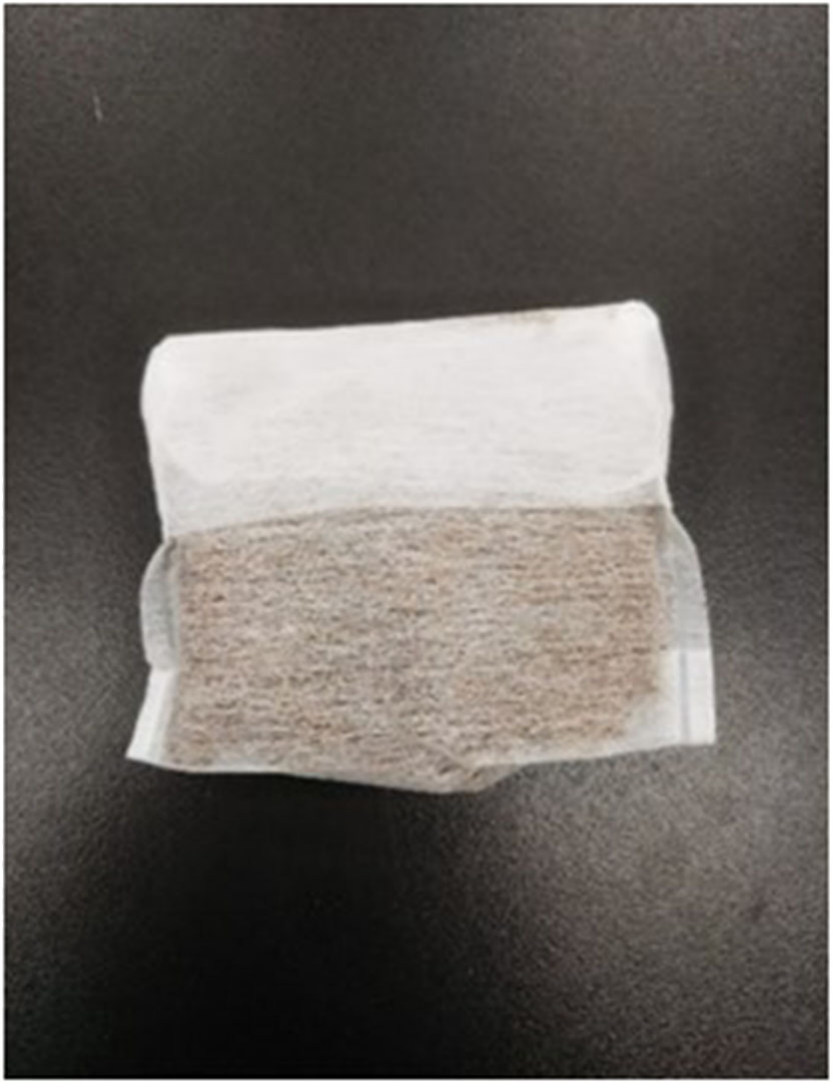
A prepared odor sachet.

**Figure 3:**
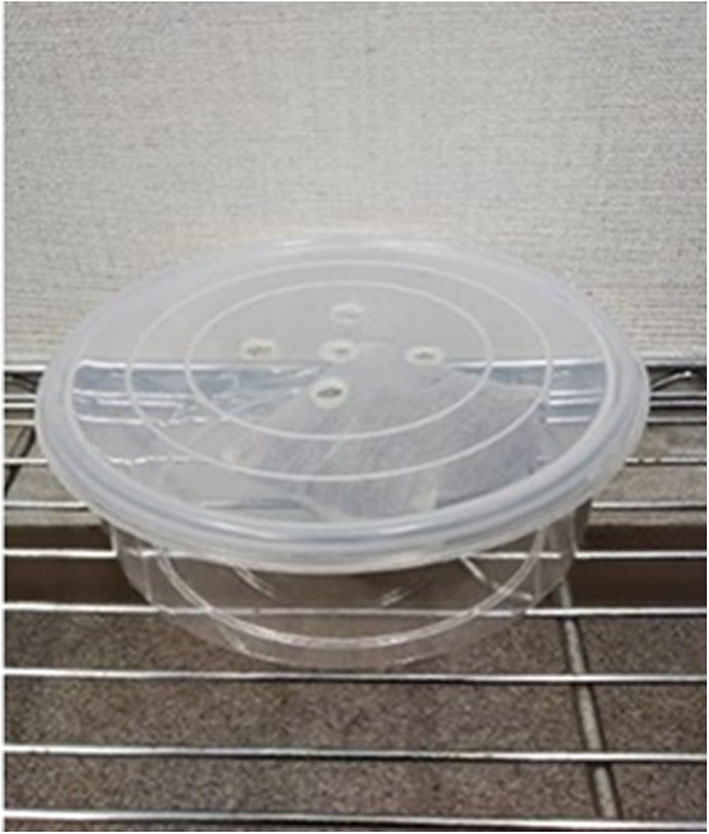
A plastic container holding an odor sachet.

Twelve male anoles captured with mealworm bait on Chichijima in July 2018 were housed in the Specified Alien Livestock Allowance Area at Nihon University in individual plastic breeding cages (W 300 mm × D 195 mm × H 205 mm) in accordance with the protocol for *Anolis* lizards (8). The lizards were unable to see each other. Each cage held a perch. The floor of each cage was covered with 37 g (5 mm deep) of home-garden coconut peat (15–30% moisture; ≤ 0.5% total N; ≤ 0.1% total phosphoric acid; ≤ 1.0% total K, pH 5.5–6.5; 70 to 90% organic matter; cation exchange capacity = 80–110 meq/100 g; maximum water capacity = 800–1000%) (Figure 1). After 72 h, the peat was recovered, sealed in a press-seal plastic bag, and stored in a freezer at −27°C. Other cages containing only peat were left in the same environment, and the peat was used to adjust concentrations and also to create the controls. The control peat also frozen. The anole population density (/ha) was calculated from the case floor area (390 cm^2^) at 256 410/ha. As the population density in the Ogasawara Islands was 1270/ha (9), we set 0.2% (512 anoles/ha) as the target odor concentration for the basic training. To prepare a test odor sachet (10 g peat), the day before the test we homogenized the 37 g from one cage and weighed out 0.02 g on an electronic balance, in addition to 9.98 g of control peat, and placed the total 10.00 g in a nonwoven cloth sachet (Marusan Industry Co., Ltd., Ehime, Japan; Figure 2). We also made up a 10 g sachet from the control peat (i.e., control sachet). These sachets were sealed in press-seal plastic bags stored at −27° C until the test. Just before the test, each sachet was sealed in a perforated plastic container (V-5, Sanoya Industry Co., Ltd. Aichi, Japan; Figure 3). The dogs were able to touch the container but not the sachet. For the test, we used 5 odor levels, setting anole densities of 385/ha (0.15%: 0.015 + 9.985 g), 256/ha (0.10%: 0.01 + 9.99 g), 128/ha (0.05%: 0.005 + 9.995 g), 26/ha (0.01%: 0.001 + 9.999 g), and 3/ha (0.001%: 0.0001 + 9.9999 g), which were prepared by the same procedure.

### Procedures

All training trials and tests were carried out in the same room (200 × 345 cm). The room held a low stainless steel table (88 cm W × 16 cm D × 15 cm H) on which the odor sachets were set. To record the dog's search behavior, we set a video camera (DCR-SR87, Sony, Tokyo, Japan) before the table and another behind it. The room temperature was controlled at around 20°C (mean, 19.63 ± 0.83°C; RH, 60.31 ± 8.64%). For both the training trials and the discrimination tests, each dog was brought from her kennel to the room.

Training used the 512/ha sachet (positive stimulus) and a control sachet. Two plastic containers containing each sachet were placed in advance with a gloved hand at 50 cm intervals on the steel table. The positive and control sachet was presented on the left and right sides equally, randomly. At the beginning of each trial, the gloved handler presented the dog with a plastic container containing the positive odor sachet to smell for up to 60 s. When the handler judged that the dog recognized the odor, she told the dog to “search” and released her. When the dog recognized the odor in the container on the table, she lay (or sat) down in front of it. The dog was given 60 s to choose. When she selected the correct container, she received a food reward. When she selected the incorrect container, the trainer ignored her for 3 min, then started next trial. Each session consisted of 20 such trials. Twenty different samples were used and changed between each session. The dog was allowed short rests both between and during sessions. One or two sessions were performed each day for 4 or 5 days per week. The number of session per a day depend on the dog's motivation. The trainer collected data from the videotapes, including the time the dog took to select the correct scent (to a precision of 0.01 s). When a dog reached a 100% success rate in one session, the two-way alternative discrimination test (e.g., 512/ha vs. control) was started. Each trial was blind. A person other than the handler set the arrangement of the container in which the odor sachet was set in advance, and the handler placed it on the table during the test. That is, the handler did not know the odor level in the container. This test starts with a population density of 512 anoles/ha. If the result of one session is >80% correct answer rate (i.e., 16/20 trials), the odor level is reduced; if it is <80%, the level is increased. That is, as the test progressed, the odor level of positive stimulus was decreased. The procedure was the same as in basic training except for odor samples. There were no situations in which no odor was presented (a blank test).

### Statistical Analysis

We compared the numbers of sessions required to reach the basic training criterion between dogs and the average search times between correct and incorrect choices in each session by Wilcoxon's signed-rank test. We examined the effect of session on the average search time of a dog's correct and incorrect choices in training by the same test. In the two-way alternative discriminative tests, we compared differences in average search times of correct or incorrect choices both between odor levels and within each odor level by Kruskal–Wallis test. When significant effects were found, we used a *post hoc* Steel–Dwass tests for pairwise comparison of the means of the search times.

## Results

### Training

The correct answer rate for the first session was different for the two dogs; with 85% (17/20) for Labrador Retriever and 95% (19/20) for German Shepherd dog. However, the total number of sessions (of 20 trials each) to reach the criterion was only 2 for both dogs.

There was no significant difference between the mean search time for two dogs when they selected the positive stimulus (sachet with odor; 15.47 ± 16.12 s), and the incorrect stimulus (control sachet; 27.47 ± 18.88 s) (*U* = 89, *P* = 0.168). On the other hand, the mean search time when a dog selected the positive stimulus (sachet with odor: LR 25.54 ± 17.80 s vs. GS 5.91 ± 3.47 s; *w* = 6.68, *P* ≤ 0.01) or the incorrect stimulus (control sachet: 35.1 ± 11.13 s vs. 4.59 s; *w* = 4.50, *P* < 0.05) differed significantly between dogs. The LR's mean correct search time in training session 2 (17.82 ± 12.86 s) was significantly shorter than that in session 1 (34.60 ± 18.50 s; *w* = 2.74, *P* < 0.05).

### Different Odor Concentrations Test

The correct rate when they selected the positive odor concentration sachet in the different odor concentration tests was 75 to 100% (Median 91.25%), and the rate of correct identification of each odor level differed between the dogs (Table 1). Dogs ranged from 100% at the highest concentration to 50 and 100 % at the lowest.

**Table 1:** Numbers and rates of correct choice for each odor level.

**Odor level (anoles/ha)**	**Mean correct % of trial**	**LR**		**GS**	
		**Correct number**	**%**	**Correct number**	**%**
		**of trial**		**of trial**	
512	100	20/20	100	20/20	100
385	95	18/20	90	20/20	100
256	92.5	17/20	85	20/20	100
128	90	16/20	80	20/20	100
26	85	15/20	75	19/20	95
3	75	10/20	50	20/20	100

The search time range when the dogs made the correct choice at any odor level was 4.72 to 60.0 s for LR and 3.19 to 20.19 s for GS. There was no effect of each odor level for two dogs' mean correct (χ^2^ = 10.48, *P*= *0.06)*/incorrect search times (χ^2^ = 3.44, *P* = *0.487)*. When the correct/incorrect search time of each odor level was compared, the incorrect search time at 26 anoles/ha was significantly longer than the correct search time (*U* = 48, *P* < 0.05) (Table 2). Each dog's correct search time differed significantly among odor levels (LR χ^2^ = 17.18, *P* < 0.0001; GS χ^2^ = 18.54, *P* < 0.0001), but not her incorrect search time (LR χ^2^ = 2.47, *P* = 0.65). There was no significant difference between the correct and incorrect search times at the same odor level by either dog.

**Table 2:** Average search time ± SD at each odor level.

			**LR**		**GS**	
**Odor level (anoles/ha)**	**Correct search time**	**Incorrect search time**	**Correct search time**	**Incorrect search time**	**Correct search time**	**Incorrect search time**
512	9.37 ± 5.44	−	12.28 ± 5.45^b^	−	6.45 ± 3.30^ab^	-
385	8.07 ± 4.95	16.65 ± 7.83	10.72 ± 5.24^b^	16.65 ± 7.83	5.68 ± 2.93^b^	-
256	9.93 ± 7.28	17.31 ± 8.56	14.65 ± 7.80^ab^	17.31 ± 8.56	5.68 ± 2.93^b^	-
128	13.84 ± 13.89	24.13 ± 13.01	23.87 ± 15.26^a^	24.13 ± 13.01	5.81 ± 2.57^b^	-
26	8.28 ± 5.70^x^	12.38 ± 8.03^y^	11.23 ± 6.30^b^	13.45 ± 7.59	5.94 ± 3.58^b^	7.02
3	14.16 ± 12.47	17.92 ± 12.14	23.44 ± 17.06^ab^	17.92 ± 11.52	9.51 ± 3.91^a^	-
Ave.	10.48 ± 9.06	17.41 ± 11.19	14.40 ± 9.86	17.79 ± 10.57	5.96 ± 3.09	7.02

*x-y: Mann-Whitney, p < 0.05*.

*a-b: Steel-Dwass, p < 0.05 within dog*.

## Discussion

Both dogs reached 100% correct discrimination in two training sessions. The average correct selection rate of two dogs for the first session was 90%. It was thought that their previous experience was advantageous. In previous training with anole excrement/ urine odor, they achieved 100% correct recognition of body odor in 27 sessions (1 session = 20 trials), and of excrement/urine odor in 2 (LR) and 4 sessions (GS). The mean correct search time for excrement/urine odor was 31.23 ± 1.38 s (LR) and 8.52 ± 0.55 s (GS). In that training, the dogs were presented with cloth that had been stored with excrement/urine mass, unlike the conditions in the new training. The accuracy of odor detection is affected by the quality and intensity of the target odor; as the anole may secrete pheromones, probably from the cloacal glands, onto the surface of feces or scats (10), discrimination between exposed peat and control peat was a relatively easy task for the two dogs.

The accuracy when they selected the positive odor concentration sachet in the different odor concentration tests was 75 to 100% (Median 91.25%). Their accuracy of 512 to 128 anoles/ha showed 90% or more, which suggested the usefulness of olfactory ability in dogs. It was reported that dogs are able to sample a variety of deer species scats 0.21 scats/km (11), to sample moose scats ~ 1.4 samples/km (6). Our test did not sample for scats, but the focus was on being able to respond to the excrement/urine odor contained in the soil. In any case, it is clear that dogs can detect a slight odor from a large area. Cristescu et al. (12) examined the use of dogs in detecting koalas (*Phascolarctos cinereus*) at low densities. They suggested that the detection ability was influenced by whether dogs were on or off the leash, but not the age of the feces (fresh or old scat). In 150 field trials, the average time to find koala scats per 100 m^2^ was 5.2 min and the accuracy was 97%. In contrast, in our controlled environment the search time was <60 s. It seems that such training can both reduce the physical burden on dogs and greatly shorten the detection effort and time. Furthermore, if it is difficult for dogs to search in the field, indoor tests are still effective. Because if done indoors, it is possible to control not only the temperature, humidity and wind direction but also environmental noise and temptation odor etc. Thus, the dog is able to focus on the odor that has to be detected.

However, differences between dogs should also be taken into consideration. Svartberg (13) found a general relationship between a bold personality and an ability to learn and perform well in tasks requiring varied training. Of course, our two dogs and handlers must have experience in order to keep high motivation and accuracy of detection. While dogs have many potentials, it is also necessary to consider how we humans can use their abilities efficiently. If we are able to confirm the detection ability of dogs indoors, then we will need to test their abilities in the field as a next step. For example, it could be a test of the effect of variable weather-ground conditions on dog abilities, an alternative discrimination test of male and female anole excretion odor, or no correct choice test. We believe that testing using soil from sites of high anole high density and at the limit of detection in the Ogasawara Islands will be useful.

## Conclusion

Dogs were able to recognize different population densities created from coconut peat used to house anoles, and they can keep high motivation during detection task, even if the target odor was low density.

## Data Availability Statement

The raw data supporting the conclusions of this article will be made available by the authors, without undue reservation.

## Ethics Statement

The animal study was reviewed and approved by The Nihon University Ethics committee (EXC17B002). Written informed consent was obtained from the owners for the participation of their animals in this study.

## Author's Note

The use of dogs for detecting invasive animal species has increased in Japan. Damage to endemic species by the Carolina anole (*Anolis carolinensis*) is expanding in the Ogasawara Islands. We hypothesized that dogs trained to recognize the anole's odor would be useful in detection work. The dog was trained in a three-way test to discriminate among native Ogasawara reptiles and the anole. The dog achieved 100% accuracy in training for body odor in 27 sessions over 14 days and for excrement/urine odor in 4 sessions over 2 days. The mean correct search time differed significantly between odors in training (*P* < 0.05), but not in the discrimination test (*P* = 0.71). The rates of the correct response in the two-way discrimination test were high (body odor, 90%; excrement/urine odor, 96%). Correction rate in the three-way test was >90%. Discrimination between transferred odor and the odorless sample in training was relatively easy. However, discrimination between odors of the same anole and between odors of the native reptiles and the Carolina anole was harder.

## Author Contributions

All authors listed have made a substantial, direct and intellectual contribution to the work, and approved it for publication.

## Conflict of Interest

The authors declare that the research was conducted in the absence of any commercial or financial relationships that could be construed as a potential conflict of interest.
